# Thulium fiber laser versus Holmium laser enucleation of the prostate: 24-month outcomes from a prospective randomized non-inferiority trial

**DOI:** 10.1007/s00345-026-06338-9

**Published:** 2026-04-29

**Authors:** Maximilian Filzmayer, Cristina Cano Garcia, Miriam I. Traumann, Matthias J. Müller, Clara Humke, Philipp C. Mandel, Luis A. Kluth, Andreas Becker, Felix K.-H. Chun, Marina Kosiba

**Affiliations:** 1 Department of Urology, Goethe University Frankfurt, University Hospital, Frankfurt am Main, Germany; 2 Department of Urology, Asklepios Paulinen Clinic, Wiesbaden, Germany; 3 Urological Center at Boxberg, Neunkirchen, Germany

**Keywords:** Lower urinary tract symptoms, Laser enucleation of the prostate, Functional outcomes, Holmium laser, HoLEP, Thulium fiber laser, ThuFLEP, Soltive™, SoLEP

## Abstract

**Purpose:**

To compare 24-month outcomes of Thulium fiber laser enucleation of the prostate (ThuFLEP) and Holmium laser enucleation of the prostate (HoLEP).

**Methods:**

A 24-month follow-up analysis of a prospective randomized trial including 150 patients (ThuFLEP *n* = 74, HoLEP *n* = 76) was conducted. Primary outcome was non-inferior International Prostate Symptom Score (IPSS) and quality of life (QoL) reduction. Secondary outcomes included urinary continence, medication use reduction, erectile and ejaculatory function, and late adverse events.

**Results:**

At 24 months, non-inferiority of ThuFLEP regarding IPSS and QoL was confirmed (*p* = 0.03). Median IPSS improved from 21 preoperatively to 3 after ThuFLEP and from 20 to 4 after HoLEP (*p* < 0.001), with no significant intergroup differences at any time point (all *p* > 0.1). Median QoL improved from 4 to 1 in both groups (*p* < 0.001), likewise without significant differences at any time point (all *p* > 0.3). Continence rates improved from 74.0% preoperatively to 90.0%, pad usage decreased from 89.8% to 25.4% and ICIQ-SF decreased from 5 to 3 points (all *p* < 0.001), without intergroup differences (all *p* > 0.1). Use of medication for lower urinary tract symptoms decreased from 89.4% to 7.7% (*p* < 0.001). Ejaculatory function declined, while ejaculation-related bother remained unchanged and erectile function improved modestly, all without intergroup differences (all *p* > 0.1). Three late adverse events occurred exclusively in the ThuFLEP group, without statistical significance (*p* > 0.1).

**Conclusion:**

ThuFLEP remains non-inferior to HoLEP for patient-reported functional outcomes at 24 months and demonstrates comparable safety, continence, medication use reduction, and sexual function results over time. Both techniques represent effective, durable and safe surgical options for treating benign prostatic obstruction.

**Trial Registration Number:**

DRKS00032699 (18.09.2023, retrospectively registered).

**Supplementary Information:**

The online version contains supplementary material available at 10.1007/s00345-026-06338-9.

##  Introduction

Laser enucleation of the prostate (LEP) is a safe and effective minimally invasive surgical treatment for bladder lower urinary tract symptoms (LUTS) due to benign prostatic hyperplasia [1, 2]. It can be performed regardless of prostate size and is associated with lower morbidity than transurethral resection of the prostate and open simple prostatectomy [1, 2]. The Thulium fiber laser (TFL) represents a novel energy source with tissue-laser interaction characteristics that differ from those of the Holmium laser [3, 4].

Available evidence suggests that TFL enucleation (ThuFLEP) achieves operative efficiency and short-term functional outcomes comparable to Holmium laser enucleation (HoLEP) [4–8]. However, most of these studies are either single-armed without comparison to the established HoLEP, retrospective, or limited to short-term outcomes [5, 9–12]. In 2023, we reported a prospective randomized trial showing that ThuFLEP was non-inferior to HoLEP for International Prostate Symptom Score (IPSS) and quality of life (QoL) at three months [13]. Similarly, Enikeev et al. reported comparable outcomes using incontinence severity at three months as the primary endpoint [4]. More recently, Elmansy et al. provided comparative data including functional outcomes at 12 months [14].

Nevertheless, evidence on clinical outcomes of ThuFLEP beyond one year remains limited [9]. To address this void, we conducted this 24-month update of our prospective randomized controlled trial.

## Patients and methods

### Data collection

This study represents a 24-month update of a prospective randomized non-inferiority trial performed at the University Hospital of the Goethe University Frankfurt [13]. It was conducted in accordance with the Declaration of Helsinki after approval by the Ethics Committee of the Faculty of Medicine at the University Hospital of the Goethe University Frankfurt (approval number E 98/21, 2021–171; June 21, 2021). All participants provided written informed consent that included long-term follow-up. Inclusion and exclusion criteria were unchanged from the index report, therefore patients with prostate volume measured by transrectal ultrasound < 30ccm or preoperative evidence of urethral stricture, prostate cancer, or neurogenic voiding dysfunction were excluded. IPSS, age, prostate volume and indwelling transurethral catheter have been used for stratified randomization into the ThuFLEP and HoLEP arm.

Surgical technique, perioperative care pathways and laser settings have been reported previously [13]. Patient reported outcome measures (PROMs) were collected preoperatively and after one, three, 12 and 24 months. They included late adverse events and reinterventions, use of LUTS medication and pads, as well as standardized validated questionnaires: IPSS, QoL, International Consultation of Incontinence Questionnaire Short Form (ICIQ-SF), International Index of Erectile Function (IIEF-5) and Male Sexual Health Questionnaire Ejaculatory Dysfunction Short Form (MSHQ-EjD-SF) [15–18]. Consistent with the index protocol, continence was defined as social continence (ICIQ-SF ≤ 4 and use of no more than one security pad per day) [13].

### Statistical analysis

Patient demographics and outcome results were compared between the ThuFLEP and HoLEP groups using Wilcoxon rank sum, Welch Two Sample *t*, Fisher’s exact and Pearson’s Chi-square test. Differences from baseline to postoperative follow-up were assessed using paired t and McNemar’s test. At 24 months, the originally defined non-inferiority framework for IPSS and QoL was applied to compared ThuFLEP to HoLEP using a one-sided Welch two-sample t-test against the prespecified margin (Δ = 2 IPSS points and 0.25 QoL points; corresponding to approximately 50% of the established minimal clinically important difference and standard deviation; one-sided α = 0.025) [13].

All statistical analyses were performed using R (R Foundation for Statistical Computing, Vienna, Austria, RRID: SCR_001905, version 4.4.1). A significance level of *p* < 0.05 with Bonferroni adjustment was applied.

## Results

### Descriptive characteristics of the study population

Between 10/21 and 10/22, 74 (49.3%) patients were randomized to receive ThuFLEP and 76 (50.7%) to receive HoLEP. Median age was 69 years, and median prostate volume was 80 ccm (Table 1). As previously reported, all preoperative characteristics did not show significant differences (all *p* > 0.3) [13].

**Table 1:** Preoperative characteristics of 150 patients randomized to Thulium fiber laser (ThuFLEP) or Holmium laser enucleation of the prostate (HoLEP); partially reported previously [13]

	*N*	Overall^a^	ThuFLEP *N* = 74 (49.3%)^a^	HoLEP *N* = 76 (50.7%)^a^	*p*-value^b^
Age [years]	150	69 (65, 75)	69 (65, 74)	69 (65, 75)	0.6
PSA [ng/ml]	134	4.7 (2.8, 7.9)	4.9 (2.8, 7.1)	4.1 (2.9, 8.7)	0.6
Prostate volume [ccm]	150	80 (63, 100)	76 (62, 95)	80 (65, 108)	0.4
ASA risk score	150				> 0.9
I/II		108 (72.0%)	53 (71.6%)	55 (72.4%)	
III/IV		42 (28.0%)	21 (28.4%)	21 (27.6%)	
Anticoagulant therapy	142	20 (14.1%)	9 (12.7%)	11 (15.5%)	0.6
IPSS	115	21 (16, 26)	22 (17, 26)	20 (16, 25)	0.5
QoL	131	4 (3, 5)	4 (3, 5)	4 (3, 5)	0.6
Continence^c^	123	91 (74.0%)	47 (77.0%)	44 (71.0%)	0.4
ICIQ-SF	119	5 (0, 11)	4 (0, 12)	6 (0, 10)	0.7
Pads used per day	88	1 (1, 1)	1 (1, 1)	1 (1, 1)	0.7
Use of at least one pad per day	88	79 (89.8%)	34 (87.2%)	45 (91.8%)	0.5
Indwelling catheter	140	34 (24.3%)	18 (25.7%)	16 (22.9%)	0.7
IIEF-5	116	10 (3, 19)	8 (2, 18)	11 (4, 19)	0.3
MSHQ-EjD-SF	112	7 (4, 10)	7 (2, 9)	8 (4, 11)	0.2
MSHQ-EjD-SF bother	105	2 (1, 4)	2 (1, 3)	2 (1, 4)	0.7
Use of LUTS medication	141	126 (89.4%)	64 (90.1%)	62 (88.6%)	0.8
Alpha-blockers and 5-alpha-reductase-inhibitors		122 (86.5%)	61 (85.9%)	61 (87.1%)	0.8
Phosphodiesterase-5-inhibitors		10 (7.1%)	7 (9.9%)	3 (4.3%)	0.3
Anticholinergics and beta-3-agonists		10 (7.1%)	6 (8.5%)	4 (5.7%)	0.7
Performed by high-volume surgeon^d^	150	85 (56.7%)	41 (55.4%)	44 (57.9%)	0.8

^a^Median (IQR), *n* (%)

^b^Wilcoxon rank sum test, Welch Two Sample *t*-test, Fisher’s exact test, Pearson’s Chi-square test

^c^Continence defined as ICIQ-SF ≤ 4 and no more than one security pad per day

^d^Five surgeons participated in the trial, including two high-volume surgeons with 751 and 433 LEP cases and three low-volume surgeons with 122, 63, and 41 LEP cases prior to trial initiation. All surgeons operated in both treatment arms with no differences in surgeon distribution between treatment arms.

### 24-month update of the primary outcome

For IPSS, non-inferiority at 24 months of ThuFLEP compared to HoLEP was achieved with a mean difference of -0.11 and an upper 95% CI of 1.79, remaining below the prespecified non-inferiority margin of 2 (*p* = 0.03; Table 2). Median IPSS significantly improved from 21 preoperatively to 4 points at 24 months follow-up (*p* < 0.001; Table 3 and S2). For QoL, non-inferiority at 24 months was also confirmed with a mean difference of -0.14 and an upper 95% CI of 0.20, remaining below the prespecified non-inferiority margin of 0.25 (*p* = 0.03; Table 2). Median QoL significantly improved from 4 points preoperatively to 1 point 24 months after operation (*p* < 0.001; Table 3 and S2). No significant differences between ThuFLEP and HoLEP were observed at any time point (all *p* > 0.1; medians at 24 months: IPSS 3 vs. 4, QoL 1 vs. 1; Table 3).

**Table 2:** Welch Two Sample t-test (one-sided α = 0.025) for non-inferiority testing regarding main functional outcomes at 24 months after Thulium fiber laser (ThuFLEP) compared to Holmium laser enucleation of the prostate (HoLEP)

	*N*	Mean difference ThuFLEP vs. HoLEP	95% CI	Non-inferiority margin (Δ)	*p*-value
IPSS	90	-0.11	-∞ – 1.79	2	0.03
QoL	92	-0.14	-∞ – 0.20	0.25	0.03

**Table 3:** Postoperative characteristics of 150 patients randomized to Thulium fiber laser (ThuFLEP) or Holmium laser enucleation of the prostate (HoLEP); partially reported previously [13]

	Time point [months]	*N*	Overall^a^	ThuFLEP *N* = 74 (49.3%)^a^	HoLEP *N* = 76 (50.7%)^a^	*p*-value^b^
IPSS	1	116	9 (5, 13)	9 (5, 15)	8 (6, 13)	> 0.9
	3	129	8 (3, 12)	9 (3, 12)	7 (4, 12)	> 0.9
	12	118	5 (2, 8)	5 (2, 10)	5 (2, 7)	0.5
	24	90	4 (2, 7)	3 (2, 7)	4 (2, 8)	0.5
QoL	1	113	2 (1, 3)	2 (1, 3)	2 (1, 3)	0.5
	3	128	1 (1, 3)	1 (0, 3)	2 (1, 3)	0.6
	12	116	1 (0, 2)	1 (0, 2)	1 (0, 2)	0.5
	24	92	1 (0, 1)	1 (0, 1)	1 (0, 1)	0.4
Continence^c^	1	116	90 (77.6%)	42 (77.8%)	48 (77.4%)	> 0.9
	3	128	105 (82.0%)	48 (78.6%)	57 (85.1%)	0.3
	12	118	112 (94.9%)	53 (94.6%)	59 (95.2%)	0.8
	24	90	81 (90.0%)	34 (85.0%)	47 (94.0%)	0.1
De novo incontinence^c^	24	90	3 (3.3%)	1 (2.5%)	2 (4.0%)	0.1
ICIQ-SF	1	117	5 (0, 9)	5 (0, 9)	6 (0, 9)	0.3
	3	118	4 (0, 9)	4 (0, 9)	4 (0, 8)	0.8
	12	118	0 (0, 5)	0 (0, 4)	0 (0, 6)	0.2
	24	89	3 (0, 5)	3 (0, 5)	0 (0, 5)	0.7
Pads used per day	1	110	1 (0, 1)	1 (0, 2)	1 (0, 1)	0.8
	3	108	1 (0, 1)	1 (0, 1)	1 (0, 1)	0.5
	12	88	0 (0, 1)	0 (0, 0)	0 (0, 1)	0.7
	24	67	0 (0, 1)	0 (0, 1)	0 (0, 1)	0.9
Use of at least one pad per day	1	110	64 (58.2%)	28 (54.9%)	36 (61.0%)	0.5
	3	108	64 (59.3%)	31 (60.8%)	33 (57.9%)	0.8
	12	88	24 (27.3%)	9 (24.3%)	15 (29.4%)	0.6
	24	67	17 (25.4%)	7 (25.0%)	10 (25.6%)	> 0.9
Indwelling catheter	1	119	1 (0.8%)	0 (0.0%)	1 (1.6%)	> 0.9
	3	122	0 (0.0%)	0 (0.0%)	0 (0.0%)	NA^d^
	12	116	0 (0.0%)	0 (0.0%)	0 (0.0%)	NA^d^
	24	92	0 (0.0%)	0 (0.0%)	0 (0.0%)	NA^d^
Late adverse events or reinterventions^e^	3–24	150	3 (2.0%)	3 (4.1%)	0 (0.0%)	0.1
Urethral strictures requiring urethrotomy			2 (1.3%)	2 (2.7%)	0 (0.0%)	0.2
Bladder neck stricture requiring intervention			1 (0.7%)	1 (1.4%)	0 (0.0%)	0.5
IIEF-5	1	33	14 (5, 20)	20 (8, 24)	10 (5, 17)	0.1
	3	63	18 (9, 23)	19 (10, 24)	18 (8, 23)	0.7
	12	71	14 (6, 22)	15 (6, 21)	14 (6, 22)	0.8
	24	56	16 (9, 20)	17 (7, 20)	15 (10, 19)	> 0.9
MSHQ-EjD-SF	1	37	2 (1, 10)	4 (1, 12)	1 (1, 8)	0.2
	3	63	3 (1, 6)	4 (1, 8)	2 (1, 5)	0.3
	12	72	3 (1, 8)	4 (1, 9)	2 (1, 6)	0.5
	24	54	1 (1, 7)	3 (1, 8)	1 (1, 5)	0.4
MSHQ-EjD-SF bother	1	36	2 (1, 3)	1 (1, 2)	3 (1, 3)	0.1
	3	61	2 (1, 3)	2 (1, 3)	2 (1, 3)	0.4
	12	70	2 (1, 3)	2 (1, 3)	2 (1, 3)	0.6
	24	59	2 (1, 3)	2 (1, 3)	3 (1, 3)	0.5
Use of LUTS medication	1	118	15 (12.7%)	7 (12.7%)	8 (12.7%)	> 0.9
	3	122	26 (21.3%)	13 (21.7%)	13 (21.0%)	> 0.9
	12	117	12 (10.3%)	7 (13.0%)	5 (7.9%)	0.4
	24	91	7 (7.7%)	1 (2.5%)	6 (11.8%)	0.1
Alpha-blockers and 5-alpha-reductase-inhibitors	1	118	3 (2.5%)	2 (3.6%)	1 (1.6%)	0.8
	3	122	2 (1.6%)	0 (0.0%)	2 (3.2%)	0.5
	12	117	3 (2.6%)	1 (1.9%)	2 (3.2%)	> 0.9
	24	91	2 (2.2%)	0 (0.0%)	2 (3.9%)	0.5
Phosphodiesterase-5-inhibitors	1	118	0 (0.0%)	0 (0.0%)	0 (0.0%)	NA^d^
	3	122	2 (1.6%)	1 (1.7%)	1 (1.6%)	> 0.9
	12	117	1 (0.9%)	0 (0.0%)	1 (1.6%)	> 0.9
	24	91	3 (3.3%)	0 (0.0%)	3 (5.9%)	0.3
Anticholinergics and beta-3-agonists	1	118	13 (11.0%)	6 (10.9%)	7 (11.1%)	> 0.9
	3	122	22 (18.0%)	12 (20.0%)	10 (16.1%)	0.6
	12	117	8 (6.8%)	6 (11.1%)	2 (3.2%)	0.1
	24	91	2 (2.2%)	1 (2.5%)	1 (2.0%)	> 0.9

^a^Median (IQR), *n* (%)

^b^Wilcoxon rank sum test, Welch Two Sample *t*-test, Fisher’s exact test, Pearson’s Chi-square test

^c^Continence defined as ICIQ-SF ≤ 4 and no more than one security pad per day

^d^not applicable because no events occurred in either treatment group

^e^All patient-reported late adverse events were grade III according to Clavien-Dindo classification. No grade I, II, IV, or V procedure-related events occurred beyond 3 months

### Secondary outcomes

Only 74.0% of the patients were continent preoperatively vs. 90.0% at 24 months after the operation. Median ICIQ-SF improved from 5 preoperatively to 3 points. Pad usage (at least one per day; Table S3) decreased from 89.8% preoperatively to 25.4% with a change of median pads per day from 1 to 0. The improvements in continence rate, ICIQ-SF and pads per day reached statistical significance only beyond 3 months postoperatively (all *p* < 0.01), while there was no statistically significant difference at one and three months after operation (all *p* > 0.1; Table S2). The majority (66.7%) of the patients with persisting postoperative incontinence were already classified as incontinent preoperatively, while de novo incontinence was rare (3.3%; Table 3). No statistically significant differences between ThuFLEP and HoLEP were observed after one, three, 12 and 24 months regarding rates of continence, ICIQ-SF, pad usage and pads per day (all *p* > 0.1).

Prior to LEP, 24.3% of patients required an indwelling catheter; none did beyond 3 months postoperatively, with rates remaining 0% at both 12 and 24 months of follow-up. Between three and 24 months, no late adverse events or reinterventions occurred in the HoLEP arm. In the ThuFLEP arm, two urethral strictures requiring urethrotomy and one bladder neck stricture requiring transurethral incision were observed, without statistically significant difference to HoLEP (all *p* > 0.1).

Preoperatively, 89.4% of patients reported use of LUTS medication vs. 7.7% at 24 months after the operation (*p* < 0.001). At baseline, alpha-blockers and 5-alpha-reductase-inhibitors predominated (86.5%), while phosphodiesterase-5-inhibitors or anticholinergics and beta-3-agonists were relatively rare (each 7.1%). After 24 months, the three drug classes were used by just 2.2%, 3.3%, and 2.2% of patients, respectively. A transient postoperative increase in the use of anticholinergics and beta-3-agonists was observed between 1 month (11.0%) and 12 months (6.8%), peaking at 3 months (18.0%, *p* = 0.02; Table S2). At no time point, statistically significant differences were observed between ThuFLEP and HoLEP with respect to the proportion of patients using LUTS medication, irrespective of whether overall LUTS medication use or the three examined drug classes were considered (all *p* > 0.1, Table 3).

Regarding sexual functional outcomes, median IIEF-5 improved from 10 preoperatively to 16. IIEF-5 did not differ significantly between ThuFLEP and HoLEP at any time point (all *p* > 0.1; median at 24 months: 17 vs. 15). Median MSHQ-EjD-SF decreased from 7 preoperatively to 2 points, while the ejaculation-associated bother domain was steady at 2 points over time. Both domains of postoperative MSHQ-EjD-SF did not differ significantly between ThuFLEP and HoLEP at any time point (all *p* > 0.1; medians at 24 months: 7 vs. 6 and 2 vs. 3).

##  Discussion

Evidence on intermediate- and long-term clinical outcomes of ThuFLEP compared to HoLEP remains limited. To fill this void, we conducted this 24-month update of our prospective randomized controlled trial and made several noteworthy observations.

First, the 24-month update revealed no significant differences in the primary outcome (IPSS and QoL) between HoLEP and ThuFLEP. Non-inferiority of ThuFLEP to HoLEP for both IPSS and QoL was still evident at 24 months. Because the two lasers have different peak absorption wavelengths in water, theoretical models predict differences in tissue penetration depth, carbonization, and heat generation [19, 20]. However, throughout the entire study period, validated questionnaire scores showed no significant differences between the two groups at any assessed time point [13]. Those findings are consistent with previous studies comparing ThuFLEP and HoLEP [4, 7, 9, 11, 12, 14]. The absolute 24-month values of IPSS (median 4) and QoL (median 1) in the ThuFLEP group were comparable to other published cohorts (4–6 for IPSS and 0.7–2.1 for QoL) at six to 36 months after ThuFLEP [5, 10–12].

Second, no differences in safety outcomes were observed after 24 months of follow-up. Three late adverse events occurred in the ThuFLEP group, whereas none were observed in the HoLEP group. Given the low event numbers, no inference regarding between-group differences can be made. Perioperative and short-term postoperative complications were reported in the initial publication of this trial and did not differ significantly between both treatment groups [13]. Discussions on potential safety differences between TFL and Holmium laser are based on the same considerations as outlined above for functional outcomes [19, 20]. Three previous clinical studies reported rates of late adverse events requiring reintervention after ThuFLEP ranging from 2.1% to 3.4%, which are comparable to the current study (4.1%) and published HoLEP cohorts [4, 7, 10–12]. In a meta-analysis, Uleri et al. found no significant association between treatment group (ThuFLEP vs. HoLEP) and the risk of bladder neck contracture or urethral stenosis [9].

Third, urinary continence outcomes improved in both treatment groups over the 24-month follow-up. The increasing proportions of continent patients (from 74.0% to 90.0%) were accompanied by a decrease of pad usage (89.8% to 25.4%) and a reduction in ICIQ-SF scores (median 5 to 3 points). Notably, the majority (66.7%) of patients with persistent incontinence postoperatively had already been incontinent before surgery, while de novo incontinence was rare (3.3%). In line with the previous comparative studies, no significant differences were observed between ThuFLEP and HoLEP at any time point regarding continence rates, ICIQ-SF scores or pad usage [4, 7, 9, 11, 14]. However, direct comparison of continence rates across LEP study cohorts remains challenging due to the lack of a standardized definition of continence. Reported rates vary considerably depending on whether continence is defined as complete absence of leakage, no or minimal pad usage, and whether validated questionnaires such as ICIQ-SF are included.

Fourth, the improvement in symptoms transferred in a decrease of LUTS medication usage from 89.4% preoperatively to 7.7% at 24 months after the operation. The pronounced reduction in pharmacological treatment reflects the well-known functional benefit of both ThuFLEP and HoLEP in relieving LUTS over the long term, thereby reducing the need for medical therapy [21]. The transient increase in anticholinergic or beta-3-agonist use during the early postoperative period likely reflects temporary irritative symptoms, which are common after LEP but tend to resolve spontaneously [4, 22, 23]. This is also supported by the fact that the incontinence outcomes (ICIQ-SF, continence rate and pad usage) showed no relevant improvement until 3 months after surgery. At no time point, statistically significant differences were observed between ThuFLEP and HoLEP with respect to the proportion of patients using LUTS medication, which suggests that both procedures are equally effective not only in improving symptom scores but also in minimizing the necessity for symptom-oriented pharmacotherapy. Direct comparison with existing ThuFLEP literature is not possible, as postoperative use of LUTS medication has not been investigated in previous studies. These findings support the durability of surgical symptom control achieved by ThuFLEP and HoLEP. The complete absence of catheter dependency beyond 3 months, compared to 24.3% preoperatively, was not surprising and further underscores the long-lasting functional benefit of LEP.

Fifth, regarding sexual functional outcomes, the study period revealed trends that were in line with previously published LEP cohorts. Median IIEF-5 score numerically increased from 10 preoperatively to 16 points at 24 months, although this change did not reach statistical significance. Erectile function showed a slight recovery compared to preoperative baseline, as has been described in multiple prior studies reporting improved or at least stable erectile function after LEP procedures [24–27]. The improvement in erectile function over time may partly reflect relief of bladder outlet obstruction, reduction of LUTS, and associated improvement in QoL [24–26]. Ejaculatory function, as assessed by the MSHQ-EjD-SF, declined from 7 to 2 points, whereas scores in the ejaculation-related bother domain remained stable. This finding is consistent with previous reports attributing postoperative retrograde ejaculation as the main contributor to ejaculatory dysfunction, while showing no effect of LEP on ejaculation-related bother [24, 28]. Importantly, no statistically significant differences between ThuFLEP and HoLEP were observed at any follow-up interval for IIEF-5 and both domains of MSHQ-EjD-SF, confirming comparable outcomes for both techniques [14].

Taken together, this study provides the first published 24-months functional outcomes of a prospective randomized controlled cohort comparing ThuFLEP and HoLEP. Non-inferiority of ThuFLEP regarding patient-reported functional outcomes, measured by IPSS and QoL, remains supported 24 months after surgery. No significant differences between ThuFLEP and HoLEP were observed regarding urinary continence, late adverse events, use of LUTS medication, or erectile and ejaculatory function. ThuFLEP and HoLEP are effective, durable and safe surgical options for the treatment of benign prostatic obstruction.

### Limitations

The primary objective of this study was to provide high-quality data on the non-inferiority of ThuFLEP compared to HoLEP over a follow-up period of up to 24 months. Nevertheless, even a prospective randomized trial has limitations. First, follow-up regarding LUTS relied exclusively on PROMs with no additional objective assessments after hospital discharge. Consequently, maximum flow rate, post-void residual volume or prostate-specific antigen serum levels are not available for this 24-months update. Therefore, conclusions regarding long-term durability are limited to patient-reported outcomes and cannot be extended to objective functional or anatomical parameters. However, the central target criterion of our investigation was the evaluation of subjective micturition function and associated quality of life. Previous evidence supports the suitability of PROMs for detecting differences in perceived outcomes of health care interventions [29]. Second, although follow-up up to 24 months was prespecified, repeated formal testing of non-inferiority beyond the primary 3-month endpoint was not defined as a separate primary objective and should be interpreted within the context of an extended follow-up. Third, the applied questionnaires did not allow differentiation between urgency, stress, and mixed urinary incontinence, as more granular instruments were not collected in this cohort. Fourth, since no assessments were available between 3 and 12 months, the precise time at which certain outcomes reached statistical significance (e.g., at 6 or 9 months) cannot be determined. Moreover, follow-up assessments were subject to partially low response rates, which may limit the robustness of certain analyses, particularly for sexual function outcomes, despite comparable missingness patterns between treatment groups. All analyses outside the prespecified non-inferiority framework are considered exploratory. Therefore, any conclusions concerning urinary continence, late adverse events, use of LUTS medication, or erectile and ejaculatory function should be interpreted with this in mind. Finally, as a tertiary referral center, selection bias towards patients with elevated surgical risks is possible, although baseline characteristics were comparable to typical LEP populations [30].

## Supplementary Information

Below is the link to the electronic supplementary material.

Supplementary Material 1

## Data Availability

The data that support the findings of this study are available from the corresponding author upon reasonable request.

## Abbreviations

ThuFLEP: Thulium fiber laser enucleation of the prostate
HoLEP: Holmium laser enucleation of the prostate
LUTS: Lower urinary tract symptoms
IPSS: International Prostate Symptom Score
QoL: Quality of life
LEP: Laser enucleation of the prostate
TFL: Thulium fiber laser
PROM: Patient reported outcome measure
ICIQ-SF: International Consultation of Incontinence Questionnaire Short Form
IIEF-5: International Index of Erectile Function
MSHQ-EjD-SF: Male Sexual Health Questionnaire Ejaculatory Dysfunction Short Form
IQR: Interquartile range
NA: Not applicable

## References

[1] Li M, Qiu J, Hou Q et al (2015) Endoscopic enucleation versus open prostatectomy for treating large benign prostatic hyperplasia: a meta-analysis of randomized controlled trials. PLoS ONE 10:e0121265 25826453 10.1371/journal.pone.0121265 PMC4380430

[2] Higazy A, Tawfeek AM, Abdalla HM et al (2021) Holmium laser enucleation of the prostate versus bipolar transurethral enucleation of the prostate in management of benign prostatic hyperplasia: a randomized controlled trial. Int J Urol 28:333–338 33327043 10.1111/iju.14462

[3] Kronenberg P, Traxer O (2019) The laser of the future: reality and expectations about the new thulium fiber laser-a systematic review. Transl Androl Urol 8:S398–s417 31656746 10.21037/tau.2019.08.01 PMC6790412

[4] Enikeev D, Taratkin M, Babaevskaya D et al (2022) Randomized prospective trial of the severity of irritative symptoms after HoLEP vs ThuFLEP. World J Urol 40:2047–2053 35690952 10.1007/s00345-022-04046-8

[5] Petov V, Babaevskaya D, Taratkin M et al (2022) Thulium fiber laser enucleation of the prostate: prospective study of mid- and long-term outcomes in 1328 patients. J Endourol 36:1231–1236 35414204 10.1089/end.2022.0029

[6] Enikeev D, Taratkin M, Laukhtina E et al (2019) En bloc and two-lobe techniques for laser endoscopic enucleation of the prostate: retrospective comparative analysis of peri- and postoperative outcomes. Int Urol Nephrol 51:1969–1974 31432393 10.1007/s11255-019-02259-2

[7] Morozov A, Taratkin M, Kozlov V et al (2020) Retrospective assessment of endoscopic enucleation of prostate complications: a single-center experience of more than 1400 patients. J Endourol 34:192–197 31810402 10.1089/end.2019.0630

[8] Elmansy H, Hodhod A, Elshafei A et al (2022) Comparative analysis of MOSES(TM) technology versus novel thulium fiber laser (TFL) for transurethral enucleation of the prostate: a single-institutional study. Arch Ital Urol Androl 94:180–185 35775343 10.4081/aiua.2022.2.180

[9] Uleri A, Long Depaquit T, Farré A et al (2024) Thulium fiber versus holmium:yttrium-aluminum-garnet laser for endoscopic enucleation of the prostate: a systematic review and meta-analysis. Eur Urol Focus 10:914–921 38897872 10.1016/j.euf.2024.06.005

[10] Kamalov AA, Sorokin NI, Dzitiev VK et al (2024) Propensity score-matched analysis comparing perioperative, functional, and safety outcomes between thulium fiber laser and bipolar enucleation of the prostate performed by a single surgeon with two years of follow-up. Investig Clin Urol 65:139–147 38454823 10.4111/icu.20230270 PMC10925738

[11] Gauhar V, Nedbal C, Castellani D et al (2024) Comparison between thulium fiber laser and high-power holmium laser for anatomic endoscopic enucleation of the prostate: a propensity score-matched analysis from the REAP registry. Eur Urol Focus 10:182–188 37414615 10.1016/j.euf.2023.06.009

[12] Romero Otero J, Justo Quintas J, García Gómez B et al (2024) Prospective randomized multicenter study to evaluate holmium vs. new thulium fiber laser for prostate enucleation. Minerva Urol Nephrol 76:491–498 39051894 10.23736/S2724-6051.24.05706-9

[13] Kosiba M, Filzmayer M, Welte MN et al (2024) Thulium fiber laser vs. holmium laser enucleation of the prostate: results of a prospective randomized non-inferiority trial. World J Urol 42:49 38244076 10.1007/s00345-023-04748-7 PMC10799774

[14] Elmansy H, Alhelal S, Blahitko O et al (2025) Thulium fiber laser versus holmium MOSES(TM) laser enucleation of the prostate for the treatment of benign prostatic hyperplasia: a randomized prospective clinical study. Prostate Cancer Prostatic Dis 29(1):152-158 10.1038/s41391-025-00996-3 40624120

[15] Rosen RC, Catania JA, Althof SE et al (2007) Development and validation of four-item version of Male Sexual Health Questionnaire to assess ejaculatory dysfunction. Urology 69:805–809 17482908 10.1016/j.urology.2007.02.036

[16] Barry MJ, Fowler FJ, O’Leary MP et al (1992) The American urological association symptom index for benign prostatic hyperplasia. J Urol 148:1549–1557 1279218 10.1016/s0022-5347(17)36966-5

[17] Rosen RC, Cappelleri JC, Smith MD et al (1999) Development and evaluation of an abridged, 5-item version of the International Index of Erectile Function (IIEF-5) as a diagnostic tool for erectile dysfunction. Int J Impot Res 11:319–326 10637462 10.1038/sj.ijir.3900472

[18] Avery K, Donovan J, Peters TJ et al (2004) ICIQ: a brief and robust measure for evaluating the symptoms and impact of urinary incontinence. Neurourol Urodyn 23:322–330 15227649 10.1002/nau.20041

[19] Fried NM, Murray KE (2005) High-power thulium fiber laser ablation of urinary tissues at 1.94 microm. J Endourol 19:25–31 15735378 10.1089/end.2005.19.25

[20] Ortner G, Rice P, Nagele U et al (2023) Tissue thermal effect during lithotripsy and tissue ablation in endourology: a systematic review of experimental studies comparing Holmium and Thulium lasers. World J Urol 41:1–12 36515722 10.1007/s00345-022-04242-6

[21] Ory J, Nackeeran S, Rainer Q et al (2022) Persistent use of medical therapy after surgery for lower urinary tract symptoms: a retrospective database analysis. World J Urol 40:169–175 34435214 10.1007/s00345-021-03819-x

[22] Kim SJ, Park SG, Pak S et al (2023) Predictive factors for postoperative medication therapy for overactive bladder symptoms after holmium laser enucleation of prostate. Int J Urol 30:1036–1043 37522563 10.1111/iju.15260

[23] Warner JN, Nunez RN, Tyson MD et al (2011) A multiinstitutional study of the effects of medical therapy for lower urinary symptoms on the perioperative outcomes of holmium laser enucleation of the prostate. Urology 78:1385–1390 21871654 10.1016/j.urology.2011.03.039

[24] Roper C, Slade A, Caras R et al (2024) Ejaculatory and erectile function outcomes following holmium laser enucleation of the prostate. Prostate 84:791–796 38558096 10.1002/pros.24697

[25] Spirito L, Capra M, Sciorio C et al (2024) Long-term functional outcomes and predictors of efficacy in thulium laser enucleation of the prostate (ThuLEP) for benign prostatic hyperplasia (BPH): a retrospective observational study. J Basic Clin Physiol Pharmacol 35:169–174 38915209 10.1515/jbcpp-2024-0036

[26] Capogrosso P, Ventimiglia E, Ferrari M et al (2016) Long-term sexual outcomes after holmium laser enucleation of the prostate: which patients could benefit the most? Int J Impot Res 28:189–193 27465782 10.1038/ijir.2016.29

[27] Enikeev D, Glybochko P, Rapoport L et al (2018) Impact of endoscopic enucleation of the prostate with thulium fiber laser on the erectile function. BMC Urol 18:87 30314492 10.1186/s12894-018-0400-1 PMC6186032

[28] Placer J, Salvador C, Planas J et al (2015) Effects of holmium laser enucleation of the prostate on sexual function. J Endourol 29:332–339 25133981 10.1089/end.2014.0502

[29] Agarwal A, Pain T, Levesque JF et al (2022) Patient-reported outcome measures (PROMs) to guide clinical care: recommendations and challenges. Med J Aust 216:9–11 34897693 10.5694/mja2.51355 PMC9299767

[30] Pallauf M, Kunit T, Ramesmayer C et al (2021) Endoscopic enucleation of the prostate (EEP). The same but different-a systematic review. World J Urol 39:2383–2396 33956196 10.1007/s00345-021-03705-6 PMC8332586

