# Supplementary material for: Thulium fiber laser versus Holmium laser enucleation of the prostate: 24-month outcomes from a prospective randomized non-inferiority trial
Source: World J Urol. 2026 Apr 29;44(1):329. doi: 10.1007/s00345-026-06338-9 (PMC13128747; doi:10.1007/s00345-026-06338-9)

**Table S1:** Means of preoperative and postoperative characteristics of 150 patients randomized to Thulium fiber laser (ThuFLEP) or Holmium laser enucleation of the prostate (HoLEP)

|  | **Time point [months]** | **N** | **Overall**^a^ | **ThuFLEP**  **N=74 (49.3%)**^a^ | **HoLEP**  **N=76 (50.7%)**^a^ | **p-value**^b^ |
| --- | --- | --- | --- | --- | --- | --- |
| **IPSS** | preoperative | 119 | 20.27 (7.34) | 20.43 (7.93) | 20.09 (6.69) | 0.8 |
|  | 1 | 116 | 9.67 (6.06) | 9.67 (6.13) | 9.67 (6.05) | >0.9 |
|  | 3 | 129 | 8.24 (6.12) | 8.35 (6.53) | 8.13 (5.75) | 0.8 |
|  | 12 | 118 | 6.30 (5.84) | 7.16 (6.99) | 5.52 (4.49) | 0.1 |
|  | 24 | 90 | 5.39 (5.11) | 5.32 (5.61) | 5.43 (4.78) | >0.9 |
| **QoL** | preoperative | 131 | 4.11 (1.44) | 4.12 (1.51) | 4.10 (1.36) | >0.9 |
|  | 1 | 113 | 2.27 (1.78) | 2.13 (1.72) | 2.38 (1.83) | 0.5 |
|  | 3 | 128 | 1.77 (1.48) | 1.68 (1.44) | 1.85 (1.51) | 0.5 |
|  | 12 | 116 | 1.20 (1.20) | 1.31 (1.33) | 1.10 (1.08) | 0.3 |
|  | 24 | 92 | 1.00 (0.94) | 0.93 (0.94) | 1.06 (0.93) | 0.5 |
| **ICIQ-SF** | preoperative | 119 | 6.11 (5.75) | 6.22 (5.85) | 6.00 (5.69) | 0.8 |
|  | 1 | 117 | 5.85 (5.44) | 5.25 (5.14) | 6.39 (5.69) | 0.3 |
|  | 3 | 118 | 5.19 (5.15) | 5.31 (5.22) | 5.07 (5.12) | 0.8 |
|  | 12 | 118 | 2.81 (4.18) | 2.21 (3.65) | 3.34 (4.57) | 0.1 |
|  | 24 | 89 | 3.16 (3.90) | 3.28 (3.90) | 3.06 (3.94) | 0.8 |
| **Pads used per day** | preoperative | 88 | 1.11 (0.61) | 1.10 (0.68) | 1.12 (0.56) | 0.9 |
|  | 1 | 110 | 1.08 (1.44) | 1.06 (1.38) | 1.10 (1.51) | 0.9 |
|  | 3 | 108 | 0.92 (1.12) | 0.96 (1.02) | 0.88 (1.21) | 0.7 |
|  | 12 | 88 | 0.41 (0.78) | 0.38 (0.76) | 0.43 (0.81) | 0.8 |
|  | 24 | 67 | 0.37 (0.74) | 0.43 (0.84) | 0.33 (0.66) | 0.6 |
| **IIEF-5** | preoperative | 116 | 11.10 (8.22) | 10.44 (8.69) | 11.68 (7.81) | 0.4 |
|  | 1 | 33 | 13.36 (8.80) | 15.75 (9.45) | 11.12 (7.76) | 0.1 |
|  | 3 | 63 | 15.48 (8.25) | 15.71 (8.69) | 15.29 (8.00) | 0.8 |
|  | 12 | 71 | 13.97 (8.24) | 14.37 (8.18) | 13.68 (8.38) | 0.7 |
|  | 24 | 56 | 14.18 (7.30) | 13.81 (8.50) | 14.40 (6.61) | 0.8 |
| **MSHQ-EjD-SF** | preoperative | 112 | 6.99 (4.27) | 6.42 (4.24) | 7.51 (4.26) | 0.2 |
|  | 1 | 37 | 5.00 (4.88) | 6.06 (5.55) | 4.00 (4.04) | 0.2 |
|  | 3 | 63 | 5.63 (13.62) | 8.37 (20.35) | 3.58 (3.26) | 0.2 |
|  | 12 | 72 | 4.40 (4.09) | 4.87 (4.30) | 4.07 (3.95) | 0.4 |
|  | 24 | 54 | 3.94 (3.87) | 4.70 (4.16) | 3.50 (3.69) | 0.3 |
| **MSHQ-EjD-SF bother** | preoperative | 105 | 2.51 (1.41) | 2.44 (1.39) | 2.58 (1.45) | 0.6 |
|  | 1 | 36 | 2.17 (1.18) | 1.67 (0.97) | 2.67 (1.19) | 0.1 |
|  | 3 | 61 | 2.31 (1.19) | 2.19 (1.20) | 2.40 (1.19) | 0.5 |
|  | 12 | 70 | 2.40 (1.36) | 2.32 (1.35) | 2.46 (1.37) | 0.7 |
|  | 24 | 59 | 2.41 (1.25) | 2.32 (1.36) | 2.46 (1.19) | 0.7 |

^a^ Mean (SD)

^b^ Welch Two Sample t-test

**Table S2:** Differences (∆) to preoperative baseline for postoperative characteristics for the overall study cohort, Thulium fiber laser (ThuFLEP) and Holmium laser enucleation of the prostate (HoLEP) patients

|  | **Time point [months]** | **Overall** | | **ThuFLEP** | | **HoLEP** | |
| --- | --- | --- | --- | --- | --- | --- | --- |
|  |  | **∆**^a^ | **p-value^b^** | **∆**^a^ | **p-value^b^** | **∆**^a^ | **p-value^b^** |
| **IPSS** | 1 | -10.60 | **<0.001** | -10.76 | **<0.001** | -10.42 | **<0.001** |
|  | 3 | -12.03 | **<0.001** | -12.08 | **<0.001** | -11.96 | **<0.001** |
|  | 12 | -13.97 | **<0.001** | -13.27 | **<0.001** | -14.57 | **<0.001** |
|  | 24 | -14.88 | **<0.001** | -15.10 | **<0.001** | -14.66 | **<0.001** |
| **QoL** | 1 | -1.84 | **<0.001** | -1.99 | **<0.001** | -1.71 | **<0.001** |
|  | 3 | -2.34 | **<0.001** | -2.44 | **<0.001** | -2.24 | **<0.001** |
|  | 12 | -2.91 | **<0.001** | -2.80 | **<0.001** | -3.00 | **<0.001** |
|  | 24 | -3.11 | **<0.001** | -3.19 | **<0.001** | -3.04 | **<0.001** |
| **Continence [%]**^c^ | 1 | +2.3 | >0.9 | -0.7 | >0.9 | +5.2 | >0.9 |
|  | 3 | +7.0 | 0.5 | -0.8 | 0.5 | +14.5 | 0.1 |
|  | 12 | +20.1 | **<0.001** | +17.6 | 0.03 | +22.7 | 0.03 |
|  | 24 | +16.0 | **<0.01** | +8.0 | 0.2 | +23.0 | 0.02 |
| **ICIQ-SF** | 1 | -0.25 | 0.9 | -0.97 | 0.3 | +0.39 | 0.3 |
|  | 3 | -0.92 | 0.5 | -0.91 | 0.9 | -0.93 | 0.4 |
|  | 12 | -3.30 | **<0.001** | -4.01 | **<0.001** | -2.66 | **<0.01** |
|  | 24 | -2.95 | **<0.001** | -2.95 | **<0.01** | -2.94 | **<0.001** |
| **Pads used per day** | 1 | -0.03 | 0.8 | -0.04 | 0.6 | -0.02 | >0.9 |
|  | 3 | -0.20 | 0.1 | -0.14 | 0.4 | -0.25 | 0.04 |
|  | 12 | -0.70 | **<0.001** | -0.72 | 0.1 | -0.69 | **<0.001** |
|  | 24 | -0.74 | **<0.001** | -0.67 | **<0.001** | -0.79 | **<0.001** |
| **Use of at least on pad per day [%]** | 1 | -31.6 | **<0.001** | -32.3 | 0.03 | -30.8 | **<0.01** |
|  | 3 | -30.5 | **<0.001** | -26.4 | 0.2 | -33.9 | **<0.01** |
|  | 12 | -62.5 | **<0.001** | -62.9 | 0.04 | -62.4 | **<0.001** |
|  | 24 | -64.4 | **<0.001** | -62.2 | **<0.01** | -66.2 | **<0.001** |
| **Indwelling catheter [%]** | 1 | -24.2 | **<0.001** | -25.7 | NA^d^ | -22.7 | **<0.01** |
|  | 3 | -25.0 | NA^d^ | -25.7 | NA^d^ | -24.3 | NA^d^ |
|  | 12 | -25.0 | NA^d^ | -25.7 | NA^d^ | -24.3 | NA^d^ |
|  | 24 | -25.0 | NA^d^ | -25.7 | NA^d^ | -24.3 | NA^d^ |
| **IIEF-5** | 1 | +2.27 | 0.6 | +5.31 | 0.8 | -0.56 | 0.3 |
|  | 3 | +4.38 | 0.1 | +5.28 | 0.6 | +3.61 | 0.1 |
|  | 12 | +2.87 | 0.7 | +3.93 | 0.8 | +2.01 | 0.8 |
|  | 24 | +3.08 | 0.3 | +3.37 | 0.8 | +2.72 | 0.4 |
| **MSHQ-EjD-SF** | 1 | -1.99 | **<0.01** | -0.36 | 0.2 | -3.51 | **<0.01** |
|  | 3 | -1.36 | **<0.001** | +1.96 | **<0.001** | -3.93 | **<0.001** |
|  | 12 | -2.59 | **<0.001** | -1.55 | **<0.01** | -3.44 | **<0.001** |
|  | 24 | -3.05 | **<0.001** | -1.72 | 0.04 | -4.01 | **<0.001** |
| **MSHQ-EjD-SF bother** | 1 | -0.35 | 0.8 | -0.77 | 0.1 | 0.08 | 0.5 |
|  | 3 | -0.20 | 0.5 | -0.25 | 0.5 | -0.18 | 0.7 |
|  | 12 | -0.11 | 0.6 | -0.12 | 0.9 | -0.12 | 0.6 |
|  | 24 | -0.11 | 0.1 | -0.12 | 0.6 | -0.12 | 0.1 |
| **Use of LUTS medication [%]** | 1 | -76.6 | **<0.001** | -77.4 | **<0.001** | -75.9 | **<0.001** |
|  | 3 | -68.1 | **<0.001** | -68.5 | **<0.001** | -67.6 | **<0.001** |
|  | 12 | -79.1 | **<0.001** | -77.2 | **<0.001** | -80.6 | **<0.001** |
|  | 24 | -81.7 | **<0.001** | -87.6 | **<0.001** | -76.8 | **<0.001** |
| Alpha-blockers and 5-alpha-reductase-inhibitors [%] | 1 | -84.0 | **<0.001** | -82.3 | **<0.001** | -85.6 | **<0.001** |
|  | 3 | -84.9 | **<0.001** | -85.9 | NA^d^ | -83.9 | **<0.001** |
|  | 12 | -84.0 | **<0.001** | -84.1 | **<0.001** | -84.0 | **<0.001** |
|  | 24 | -84.3 | **<0.001** | -85.9 | NA^d^ | -83.2 | **<0.001** |
| Phosphodiesterase-5-inhibitors [%] | 1 | -7.1 | NA^d^ | -9.9 | NA^d^ | -4.3 | NA^d^ |
|  | 3 | -5.5 | 0.1 | -8.2 | 0.1 | -2.7 | 0.6 |
|  | 12 | -6.2 | 0.03 | -9.9 | NA^d^ | -2.7 | 0.6 |
|  | 24 | -3.8 | 0.5 | -9.9 | NA^d^ | +1.6 | >0.9 |
| Anticholinergics and beta-3-agonists [%] | 1 | +3.9 | 0.1 | +2.5 | 0.4 | +5.4 | 0.3 |
|  | 3 | +10.9 | 0.02 | +11.5 | 0.1 | +10.4 | 0.1 |
|  | 12 | -0.3 | 0.8 | +2.7 | 0.4 | -2.5 | >0.9 |
|  | 24 | -4.9 | 0.4 | -6.0 | >0.9 | -3.8 | 0.6 |

^a^ Mean difference, proportion difference

^b^ Paired t-test, McNemar's Chi-squared test

^c^ Continence defined as ICIQ-SF≤4 and no more than one security pad

^d^ not applicable because no events occurred in treatment group.

**Figure S1:** IPSS (International Prostate Symptom Score), QoL (quality of life), indwelling catheter, continence, International Consultation of Incontinence Questionnaire Short Form (ICIQ-SF), use of at least one pad per day and use of LUTS medication preoperative (preop) and after one, three, 12 and 24 months (M) of 150 patients randomized to Thulium fiber laser (ThuFLEP, red) or Holmium laser enucleation of the prostate (HoLEP, blue)

**Table S3:** Detailed breakdown of preoperative and postoperative pad use per day of 150 patients randomized to Thulium fiber laser (ThuFLEP) or Holmium laser enucleation of the prostate (HoLEP), supplement to Table 1 and 3

| **Time point [months]** | **Pad use per day** | **N** | **Overall**^a^ | **ThuFLEP**  **N=74 (49.3%)**^a^ | **HoLEP**  **N=76 (50.7%)**^a^ | **p-value**^b^ |
| --- | --- | --- | --- | --- | --- | --- |
| preoperative | 0 | 88 | 9 (10.2%) | 5 (12.8%) | 4 (8.2%) | 0.8 |
|  | 1 |  | 63 (71.6%) | 27 (69.2%) | 36 (73.5%) |  |
|  | >1 |  | 16 (18.2%) | 7 (17.9%) | 9 (18.4%) |  |
| 1 | 0 | 108 | 46 (42.6%) | 23 (46.0%) | 23 (39.7%) | 0.7 |
|  | 1 |  | 41 (38.0%) | 17 (34.0%) | 24 (41.4%) |  |
|  | >1 |  | 21 (19.4%) | 10 (20.0%) | 11 (19.0%) |  |
| 3 | 0 | 105 | 44 (41.9%) | 20 (40.0%) | 24 (43.6%) | 0.3 |
|  | 1 |  | 44 (41.9%) | 19 (38.0%) | 25 (45.5%) |  |
|  | >1 |  | 17 (16.2%) | 11 (22.0%) | 6 (10.9%) |  |
| 12 | 0 | 87 | 64 (73.6%) | 28 (75.7%) | 36 (72.0%) | 0.8 |
|  | 1 |  | 17 (19.5%) | 6 (16.2%) | 11 (22.0%) |  |
|  | >1 |  | 6 (6.9%) | 3 (8.1%) | 3 (6.0%) |  |
| 24 | 0 | 67 | 50 (74.6%) | 21 (75.0%) | 29 (74.4%) | 0.3 |
|  | 1 |  | 11 (16.4%) | 3 (10.7%) | 8 (20.5%) |  |
|  | >1 |  | 6 (9.0%) | 4 (14.3%) | 2 (5.1%) |  |

^a^ n (%)

^b^ Pearson's Chi-square test

**Figure S2:** CONSORT-style flow diagram


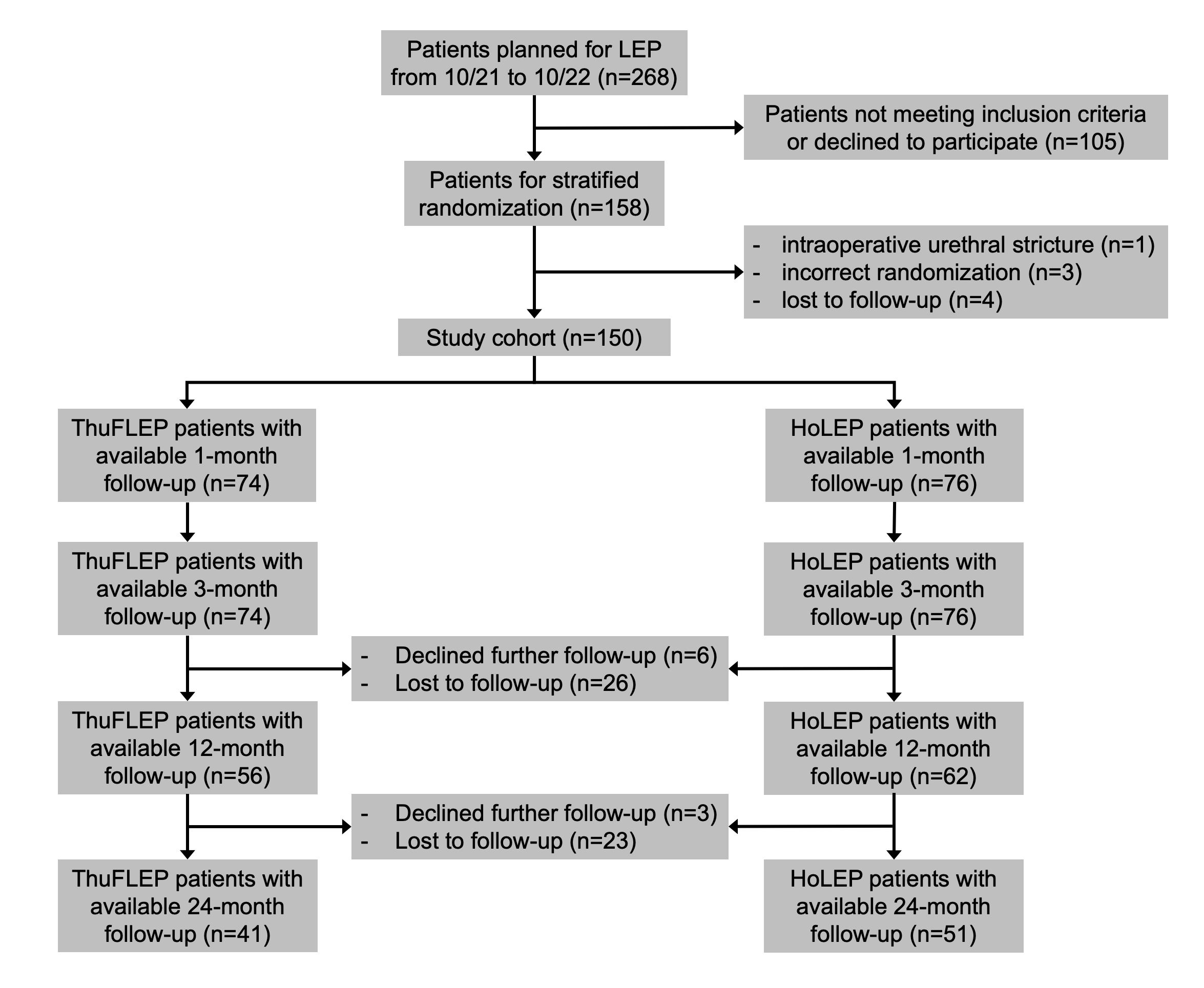

Supplement: Supplementary file 1 — Supplementary Material 1 [file 345_2026_6338_MOESM1_ESM.docx]
